# Intracellular Complement Activation Sustains T Cell Homeostasis and Mediates Effector Differentiation

**DOI:** 10.1016/j.immuni.2013.10.018

**Published:** 2013-12-12

**Authors:** M. Kathryn Liszewski, Martin Kolev, Gaelle Le Friec, Marilyn Leung, Paula G. Bertram, Antonella F. Fara, Marta Subias, Matthew C. Pickering, Christian Drouet, Seppo Meri, T. Petteri Arstila, Pirkka T. Pekkarinen, Margaret Ma, Andrew Cope, Thomas Reinheckel, Santiago Rodriguez de Cordoba, Behdad Afzali, John P. Atkinson, Claudia Kemper

**Affiliations:** 1 Department of Medicine, Division of Rheumatology, Washington University School of Medicine, Saint Louis, MO 63110, USA; 2 MRC Centre for Transplantation, Division of Transplant Immunology and Mucosal Biology, King’s College London, Guy’s Hospital, London SE1 9RT, UK; 3 Departamento de Immunología, Centro de Investigaciones Biológicas, Consejo Superior de Investigaciones Científicas, Madrid 28006, Spain; 4 Centre for Complement and Inflammation Research, Imperial College, London SW7 2AZ, UK; 5 Université Joseph Fourier, GREPI/AGIM CNRS FRE3405, Grenoble, F-38041, France; 6 Haartman Institute and Research Programs Unit, Immunobiology, University of Helsinki, Helsinki, FI-00014, Finland; 7 Biomedical Research Centre, King’s Health Partners, Guy’s Hospital, London SE1 9RT, UK; 8 Academic Department of Rheumatology, King’s College London, London SE1 9RT, UK; 9 Institute of Molecular Medicine and Cell Research, and BIOSS Centre for Biological Signaling Studies, University of Freiburg, Freiburg, D-79104, Germany

## Abstract

Complement is viewed as a critical serum-operative component of innate immunity, with processing of its key component, C3, into activation fragments C3a and C3b confined to the extracellular space. We report here that C3 activation also occurred intracellularly. We found that the T cell-expressed protease cathepsin L (CTSL) processed C3 into biologically active C3a and C3b. Resting T cells contained stores of endosomal and lysosomal C3 and CTSL and substantial amounts of CTSL-generated C3a. While “tonic” intracellular C3a generation was required for homeostatic T cell survival, shuttling of this intracellular C3-activation-system to the cell surface upon T cell stimulation induced autocrine proinflammatory cytokine production. Furthermore, T cells from patients with autoimmune arthritis demonstrated hyperactive intracellular complement activation and interferon-γ production and CTSL inhibition corrected this deregulated phenotype. Importantly, intracellular C3a was observed in all examined cell populations, suggesting that intracellular complement activation might be of broad physiological significance.

## Highlights

• Complement C3 is activated intracellularly in human T cells by cathepsin L
• Intracellular C3 activation mediates cell survival and Th1 induction
• Increased intracellular C3 activation underlies T effector dysregulation in arthritis
• Patients with serum C3-deficiency retain intracellular C3a generation

## Graphical Abstract

**Figure:**
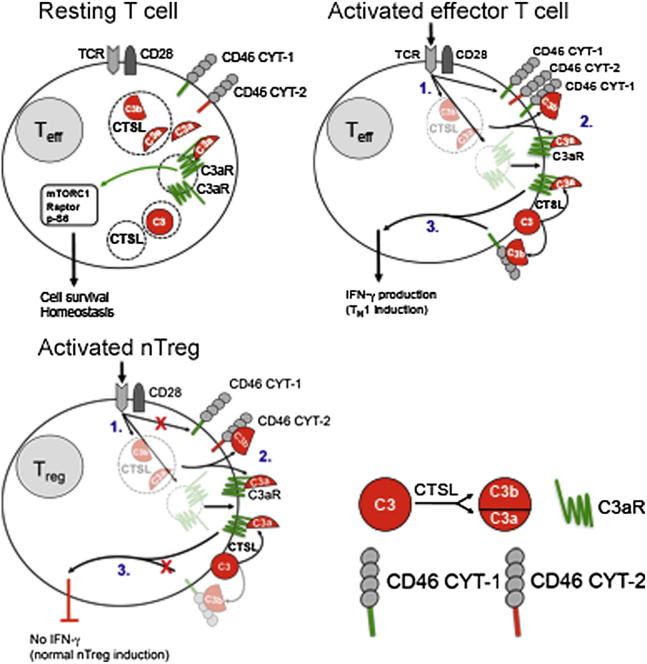

## Introduction

The complement system is a quintessential part of innate immunity and key in the protection against infections (Volanakis, 1998). As a doctrine, complement is viewed as a systemic, serum effector system, with the liver producing the majority of soluble complement proteins (Walport et al., 2001a, 2001b). Although liver-generated circulating C3 and C5 are indisputably required for the detection and removal of pathogens (Walport et al., 2001a, 2001b), an emerging paradigm suggests that immune cell-derived and intrinsically operating complement activation fragments are key in driving and modulating adaptive T cell immunity (Heeger and Kemper, 2012; Kolev et al., 2013). A growing body of evidence demonstrates the critical role of signals transduced by complement receptors expressed on CD4^+^ T cells, in addition to T cell receptor (TCR) activation, costimulation, and environmental presence of interleukin-12 (IL-12) (Murphy and Stockinger, 2010), in T helper 1 (Th1) cell-mediated immunity (Liu et al., 2005; Strainic et al., 2008). In particular, the C3 activation fragments C3a and C3b, generated by the T cell itself (Cardone et al., 2010; this study did not define the mechanism underlying autocrine C3 activation), are required for the induction of interferon-γ (IFN-γ) secretion via autocrine engagement of their respective receptors, the G protein-coupled receptor (GPCR) C3a receptor (C3aR) and the complement regulator CD46 (which binds C3b) (Le Friec et al., 2012; Liszewski et al., 2005). This observation is underpinned by the fact that CD46-deficient patients throughout life or C3-deficient patients in early childhood suffer from recurrent infections and have severely reduced T helper 1 (Th1) cell-mediated responses (Th2 cell responses are normal) (Ghannam et al., 2008; Le Friec et al., 2012). Although studies using T cells from *C3ar1*^–/–^ and *C5ar1*^–/–^ animals demonstrate that these anaphylatoxin receptors are also needed for effector cytokine secretion in mice (Strainic et al., 2008), rodents lack CD46 expression on somatic tissue (Tsujimura et al., 1998) and no functional homolog has been identified yet (Fernández-Centeno et al., 2000). This suggests that complement-mediated signaling pathways contributing to Th1 cell induction differ between mice and humans. Furthermore, the underlying mechanisms of cell-derived local complement activation are poorly understood and controversial. In the mouse system, local generation of C3 activation products observed after T cell stimulation is thought to be dependent on the formation of the C3-cleaving C3 convertase. That is, TCR and CD28 activation on mouse CD4^+^ T cells induces the expression and secretion of C3, factor B and factor D proteins, the subsequent assembly of a C3 convertase, and then extracellular cleavage of C3 (Lalli et al., 2008; Strainic et al., 2008). Because our observation that human T cells generated C3a and C3b rapidly (within 5 min) after activation does not align with the timeline for de novo induction of the required protein machinery, we hypothesized that C3 cleavage is achieved in a distinct fashion and set out to determine the mechanism of C3 activation in human CD4^+^ T cells.

Here, we have demonstrated that the rapid human CD4^+^ T cell-derived generation of C3a and C3b was mediated via a C3 convertase-independent but cathepsin L (CTSL)-dependent mechanism. We further show that complement activation occurred intracellularly where it contributed to the regulation of cell survival. In vivo significance for an essential role of intracellular C3 activation in controlling T cell activity was supported by the observations that T cells from autoimmune arthritis patients displayed increased intracellular C3a generation, mTOR activity, and proinflammatory cytokine production and that this phenotype could be reversed by the pharmacological targeting of intracellular CTSL activity.

## Results

### CTSL Cleaves Human C3 into Biologically Active C3a and C3b

**Figure 1:**
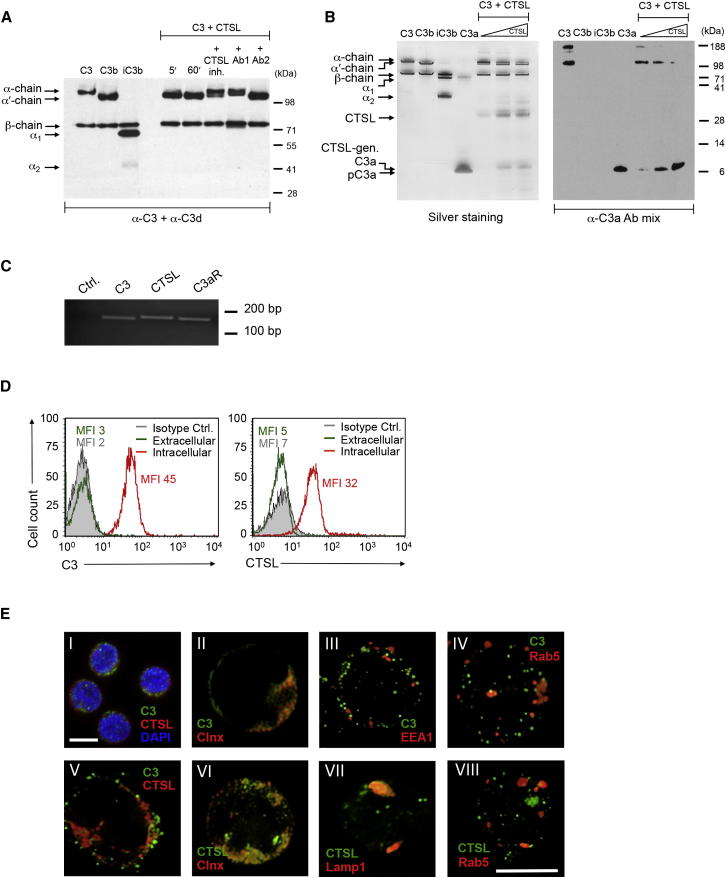
Resting Human CD4^+^ T Cells Contain Stores of C3 and C3-Activating Cathepsin L (A) CTSL-mediated C3 cleavage in the presence of CTSL inhibitors, including a chemical inhibitor, a function-blocking (Ab1) and a non-function-blocking antibody to CTSL (Ab2) and analysis by immunoblotting for C3 cleavage components. (B) CTSL-mediated cleavage of C3 generates C3a. C3 incubation with CTSL (50, 100, and 200 ng) generates C3a detected by silver staining (left panel) and immunoblotting with two anti-C3a antibodies to cleaved C3a and C3a contained in the C3 α chain (right panel). (C) T cells contain mRNAs coding for C3, CTSL, and the C3a receptor (C3aR). Data shown in (A)–(C) are representative of three experiments (n = 3). (D) Resting CD4^+^ T cells contain intracellular C3 and CTSL as assessed by flow cytometry analysis. (E) Subcellular localization of C3 and CTSL stores assessed by confocal microscopy. Scale bars represent 10 μM. (D and E) are representative of n = 5 experiments. Clnx, calnexin, endoplasmic reticulum marker; EEAI, early endosomal vesicle marker; Lamp1, lysosomal marker; MFI, mean fluorescence intensity; Rab5, late endosomal vesicle marker. (I, magnification ×60; II to VII, magnification ×100). See also Figure S1.

To explore potential C3 convertase-independent mechanisms of autocrine complement activation in human CD4^+^ T cells, we performed gene expression studies on these cells with focus on endogenous proteases. This approach revealed the presence of large amounts of messenger RNAs (mRNAs) coding for the endosomal and lysosomal proteases cathepsin B (CTSB), cathepsin G (CTSG), and CTSL in resting T cells that increased upon activation (data not shown). When we assessed the ability of these proteases to cleave C3 in vitro, we found that CTSL cleaved C3 efficiently into C3a and C3b (Figures 1A and 1B), whereas CTSG (see Figures S1A and S1B available online) and CTSB (data not shown) degraded C3 but did not generate specific activation fragments. C3 had previously been described as a substrate of CTSL expressed by human melanoma cells but in those studies, C3 was not processed into C3a and C3b but rather fully degraded (Frade et al., 1998). CTSL-mediated C3a and C3b generation could be inhibited by a CTSL-specific chemical inhibitor and by one function-blocking antibody to CTSL (Ab1, Figure 1A). Although CTSL cleaved C4 into C4a and C4b-like fragments (Figure S1C), it did not cleave C5 into C5a and C5b activation fragments (Figure S1D). Because a receptor for C4a has not been identified in any species and a role for C3a in human T cell biology is indicated, we subsequently focused on the role of CTSL-mediated C3 activation. The cleavage of C3 by CTSL created biologically active fragments as CTSL-generated C3a could induce oxidative bursts in neutrophils, and CTSL-generated C3b was able to bind CD46 (Figures S1E–S1G). We next assessed the expression of C3 and CTSL in human CD4^+^ T cells. We detected mRNA and also unanticipated C3 and CTSL protein amounts in resting T cells (Figures 1C and 1D). CTSL was found, as expected, in the endoplasmic reticulum (ER), the lysosomes, and late ER-derived secretory vesicles (Reiser et al., 2010) (Figure 1E). C3 localized also to the ER and additionally to early and late ER-derived secretory vesicles (Figure 1E). Thus, CTSL and C3 reside in overlapping and also distinct locations in resting T cells.

### CTSL Mediates Intracellular and Extracellular C3 Activation in T Cells

**Figure 2:**
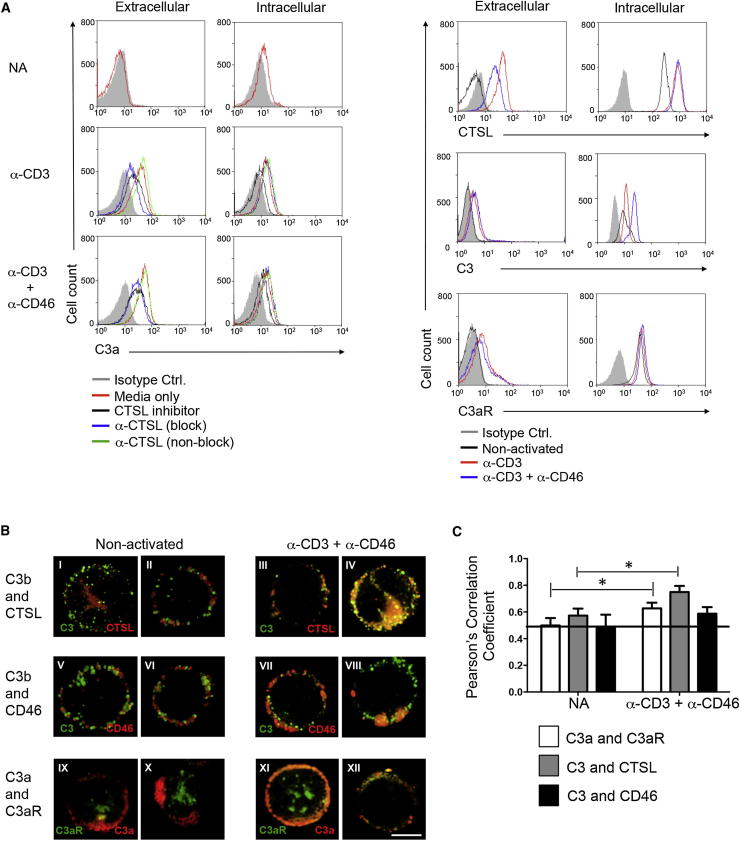
CTSL Generates Intracellular and Extracellular C3a (A) C3a generation in resting and activated T cells (1 hr) in the presence of different CTSL-blocking reagents: a chemical CTSL inhibitor (CTSLi), a function-blocking (block), and a non-function-blocking antibody to CTSL (non-block) (left panel). Expression of CTSL, C3b, and C3aR was also measured but without addition of CTSL-blocking reagents (right panels). Shown are representative data of three independently performed experiments (n = 3). (B and C) C3b and C3a and their respective receptors translocate and colocalize upon T cell activation. Nonactivated or anti-CD3 and anti-CD46-activated T cells, permeabilized and stained for C3, CTSL, C3a, C3aR, and CD46 in the combinations depicted and analyzed by confocal microscopy (B). Shown are two representative staining examples side-by-side for each condition from eight similarly performed experiments with a different donor each time (n = 8). Scale bar represents 5 μM. (C) Statistical analysis ± SD for colocalization events of the proteins assessed under (B) with Pearson’s Correlation Coefficient method. (Magnification ×100). ^∗^p < 0.05. See also Figure S2.

To address whether CTSL-mediated processing of C3 occurs in human CD4^+^ T cells, we assessed for presence of extracellular and intracellular C3a in resting T cells and in T cells that had been activated with antibodies to CD3 and CD3^+^CD46 for 1 hr. For this, an antibody to C3a was used that only recognizes a C3a neo-epitope on the cleaved fragment, but not on C3a within the C3 α chain (Hartmann et al., 1993; Miller et al., 2012). While nonactivated T cells constitutively express CD46 (Cardone et al., 2010), no C3a, CTSL, C3b, or C3aR could be found on the surface of resting T cells (Figure 2A). Upon CD3 or CD3^+^CD46 activation, however, C3a appeared on the exterior of the cell, and this C3a generation could be significantly decreased (about 20% for CD3-activation and 50% for CD3^+^CD46 activation) by addition of the CTSL inhibitor or an antibody that blocks CTSL-mediated cleavage of C3 (Figure 2A). Similarly, CTSL, C3b, and the C3aR were also detected on the surface of stimulated T cells (Figure 2A). We observed intracellular expression of the C3aR (in line with presence of *C3AR1* mRNA, Figure 1C) and a C3a generation in resting T cells. A further increase in intracellular C3a upon activation could only be prevented by the cell-permeable CTSL inhibitor, but not by the cleavage-blocking antibody (Figure 2A; for a summary of MFI values obtained, see Figure S2). In line with the presence of C3a in resting T cells, immunoblot analyses of lysates from nonactivated T cells showed predominantly the processed α chain of C3, indicative of C3b generation (Figure S2B). Confocal microscopy combined with statistical analysis of protein colocalization coefficients suggested that C3 or C3b and CTSL, C3a and C3aR, and C3 or C3b and CD46 reside in part in overlapping locations in resting T cells. Furthermore, their colocalization was increased upon T cell activation, particularly on the cell surface (Figures 2B and 2C). These data support a model in which CTSL generates “tonic” C3a from existing C3 pools in resting T cells, as well as on the cell surface upon TCR stimulation. In agreement with this, CTSL is functionally active at both an acidic pH in the lysosome, as well as pH 7.4 as occurs in an extracellular environment (Dehrmann et al., 1995). Importantly, surface translocation of this system is independent of costimulation because CD46 (Figure 2A) or CD28 (data not shown) engagement was not required.

### CSTL-Mediated Intracellular C3a Generation Is Required for T Cell Survival

**Figure 3:**
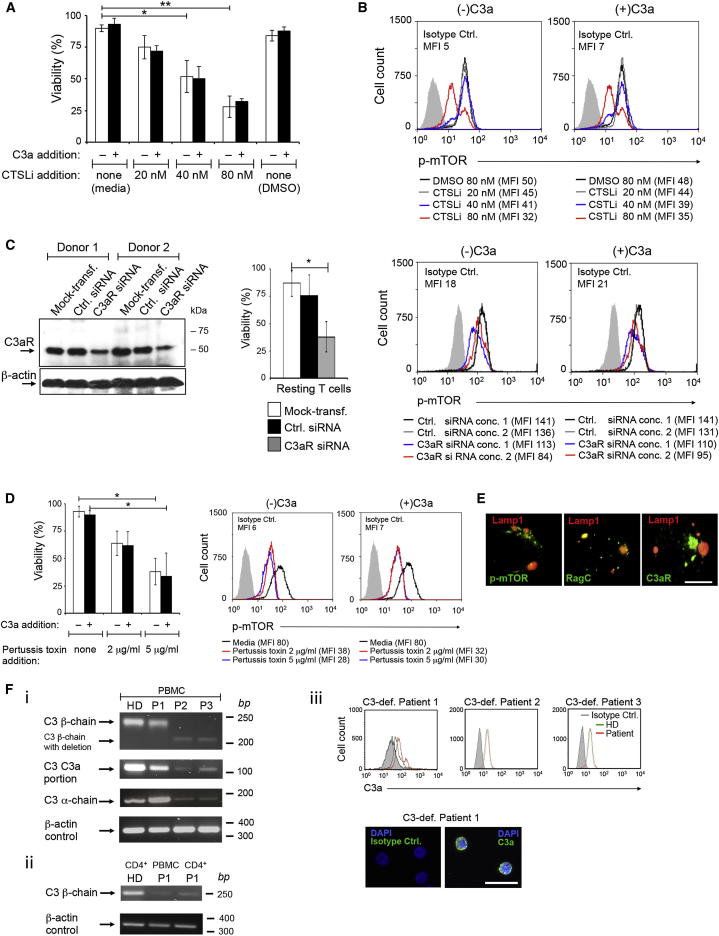
CD4^+^ T Cell Survival Is Dependent on CTSL-Mediated C3 Processing and Intracellular C3aR Signaling (A and B) Viability (A) and mTOR activation (B) in resting T cells with increasing amounts of CTSL inhibitor with or without concurrent addition of C3a (100 ng/ml) at 12 hr postaddition of reagents. (C) Effect of C3aR-specific siRNA (left panel) on viability (middle panel) and mTOR activity (right panel) in nonactivated T cells 24 hr after transfection. (D) Effect of GPCR inhibition with pertussis toxin on viability (left panel) and mTOR activation (right panel) with and without C3a addition. Data ± SD shown are derived from three independently performed experiments (n = 3). (E) Confocal microscopy analysis for C3aR, mTOR, and RagC expression and colocalization in resting CD4^+^ T cells (n = 2). Scale bar represents 5 μM. (F) *C3* mRNA and intracellular C3a protein in cells from serum C3-deficient patients. (Fi) *C3* mRNA in peripheral blood mononuclear cells (PBMCs) from three serum C3-deficient patients (P1, P2 and P3) and a healthy donor (HD). (Fii) Comparison of *C3* mRNA between PBMCs and CD4^+^ T cells from P1. Other C3 primer pairs gave similar results (data not shown). (Fiii) Activated C3 (intracellular C3a) in purified CD4^+^ T cells from C3-deficient Patients 1, 2, and 3. CD4^+^ T cells from Patient 1 were also assessed for C3a presence by confocal microscopy (two panels below). Scale bar represents 25 μM. Data (±SD) are from three experiments (n = 3) in (A)–(D). (E) ×100 magnification and (Fiii, lower panels) ×60 magnification. Conc., concentration; Ctrl. siRNA, control siRNA; Mock-transf., mock-transfected. ^∗^p < 0.05; ^∗∗^p < 0.005. See also Figure S3.

We noticed that CD4^+^ T cells cultured with increasing amounts of CTSL inhibitor (which prevented intra- and extracellular C3a generation) entered an apoptotic state within 8–12 hr (Figure 3A). C3aR (and C5aR) engagement on TCR- and CD28-stimulated mouse CD4^+^ T cells is connected with mTOR activity, which is required for T cell survival and induction of effector T cell responses (Strainic et al., 2008, 2013; Yang and Chi, 2012). In agreement with this, CTSL inhibition reduced mTOR phosphorylation in resting (Figure 3B) as well as in activated T cells (Figure S3A). Cell viability and mTOR activation could not be “rescued” by addition of purified C3a (Figures 3A and 3B). Reduction of intracellular C3aR expression via siRNA in resting T cells induced a comparable decrease in cell viability and mTOR activity (Figure 3C), as did the inhibition of GPCR signaling via addition of pertussis toxin (Figure 3D)—and neither phenotype could be reversed via C3a supplementation (Figures 3C and 3D). That the CTSL inhibitor was not toxic but exerted specific effects on CD4^+^ T cell intracellular C3a production was demonstrated in human lung epithelial cells (which also contain C3a stores). Addition of up to 1,000 times higher concentrations of CTSL inhibitor did not alter intracellular C3a generation or cell viability (Figures S3B and S3C). Furthermore, incubation of CD4^+^ T cells with a specific CTSG inhibitor had no effect on intracellular C3a amounts or cell survival (Figures S3D and S3E). Together, these results indicate that intracellular C3a generation and C3aR activation contribute to homeostatic mTOR activity and T cell survival. Indeed, a recent study demonstrated that GPCR signaling is not confined to the plasma membrane but also occurs from intracellular compartments (Irannejad et al., 2013) and C3aR expression was detected in the lysosomes of resting T cells (Figure 3E). Thus, nonactivated CD4^+^ T cells do not express C3aR on the cell surface but have strong intracellular C3aR expression and the intracellular C3aR-C3a interaction contributes to a metabolic pathway required for cell survival.

However, this raises the question of why T cells from C3-deficient patients, which cannot produce IFN-γ (Ghannam et al., 2008; Le Friec et al., 2012), survive and proliferate normally. Interestingly, although C3 serum-deficiency has been confirmed in all published case studies; whenever intracellular C3 was assessed in these patients, C3 or C3-like protein products can be observed (Ghannam et al., 2008; Katz et al., 1994; S Reis et al., 2006; Singer et al., 1994). This supports our findings: We analyzed *C3* gene mRNA expression in peripheral blood mononuclear cells (PBMCs) isolated from three C3-deficient patients (P1, P2 and P3) (Ghannam et al., 2008; Pekkarinen et al., 2013) and showed that PBMCs and CD4^+^ T cells from P1 and healthy donors had comparable *C3* mRNA patterns and amounts (Figures 3Fi and 3Fii). Cells from Patients P2 and P3 (siblings) contained reduced amounts of *C3* mRNA and also had a deletion of about 30–40 bases in the mRNA coding for the β chain, whereas mRNAs coding for the C3a portion and the α chain were of correct size (Figure 3Fi). Importantly, CD4^+^ T cells from all three patients contained normal amounts of C3a protein (Figure 3F), indicating “successful” generation of an intracellular protein form of C3 that can generate functional C3a in T cells and PBMCs in these individuals. Thus, our data suggest a potential disconnection between plasma C3 (which is chiefly derived from liver cells) and intracellular C3 presence in cases of genetically-based C3 protein deficiency. We speculate that cells from serum C3-deficient patients do not secrete C3 or C3 fragments but can generate sufficient intracellular C3a for cell survival and that, thus, combined extra- and intracellular C3 deficiency might possibly not exist in humans.

### Surface Engagement of C3aR and CD46 Drives Effector Function in Human CD4^+^ T Cells

**Figure 4:**
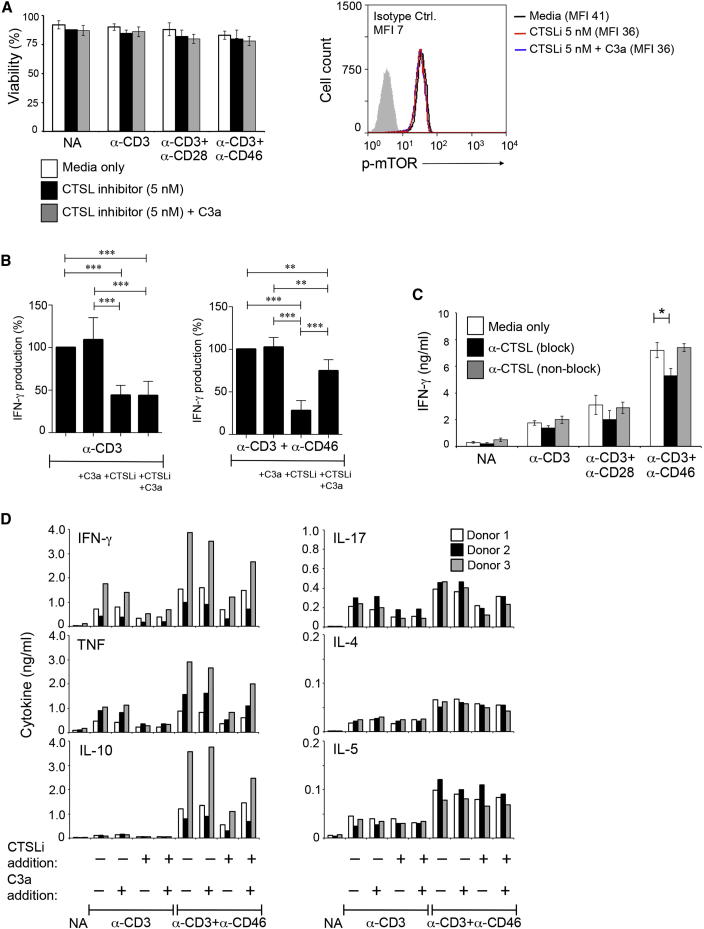
Th1 and Th17 Cell Induction Requires Cell Surface Activation of CD46 and C3aR by CTSL-Generated C3 Activation Fragments (A and B) C3a supplementation rescues CTSL inhibitor (CTSLi)-mediated diminution of Th1 cell induction. CD4^+^ T cells were stimulated as shown in media with 5 nM CTSLi with or without addition of C3a (100 ng/ml). (A) 5 nM CTSL inhibitor leaves cell viability (see Figure S4B) and mTOR activity (right panel) unaffected. (B) IFN-γ production by cells activated under these conditions was measured 36 hr after activation with IFN-γ production by CD3- (left panel) or CD3^+^CD46-activated cells (right panel) set at 100%. (C) Effect of anti-CTSL function-blocking or function-non-blocking antibodies on Th1 cell induction. (D) Effects of CTSL inhibition on Th2 and Th17 cell-mediated responses. Experiments were performed as described under (A) and (B) and production of indicated cytokines measured 36 hr after activation. Data ± SD are from five independent experiments (n = 5) in (A)–(C) and from three independent experiments in (D) with results shown as mean values of conditions performed in duplicate (n = 3). See also Figure S4.

For the induction of proinflammatory T cell effector function, TCR activation and extracellular or cell surface generation of C3a and C3b by CTSL is required. If we activated T cells in the presence of a CTSL inhibitor concentration that did not affect cell viability and mTOR phosphorylation (Figure 4A) but reduced cell surface C3a (data not shown), IFN-γ secretion was decreased by at least 50% and could be restored to about 75% by concurrent addition of C3a to the media and CD46 engagement (which mimics C3b generation) (Figures 4B and S4A). Similarly, addition of the antibody to CTSL that blocks extracellular C3a cleavage reduced IFN-γ production significantly but had no effect on cell viability (Figure S4B), whereas addition of the antibody that did not inhibit CTSL-mediated cleavage of C3 to cultures had no effect on IFN-γ (Figure 4C). As expected, C4a addition during T cell activation failed to rescue the CTSL inhibitor-mediated reduction in IFN-γ secretion (Figure S4C).

These data support a revised model for induction of T cell effector function. That is, TCR activation induces translocation of intracellular stores of C3aR to the cell surface, amplifies intracellular CTSL-mediated generation of C3a and C3b, and induces cell surface CTSL-mediated C3 activation. Extracellular C3aR and CD46 engagement by C3a and C3b subsequently supports induction of T cell effector function (Figures 2A–2C). This model is consistent with the lack of Th1 cell responses observed previously in CD46- and C3-deficient patients (Le Friec et al., 2012). Furthermore, inhibition of autocrine CTSL-mediated C3 activation not only decreased secretion of the Th1 cell-associated cytokines IFN-γ and TNF but also reduced IL-17 production by activated T cells, whereas Th2 cell-associated cytokines IL-4 and IL-5 were less affected (Figure 4D). Although IL-10 was initially classified as Th2 cell-specific cytokine, IL-10 production from CD4^+^ T cells in humans is now more strongly connected with Th1 (and Th17) cell contraction, which is marked by the cosecretion of immunosuppressive IL-10 (Cardone et al., 2010; Trinchieri, 2007). However, our data here are generated in vitro, and to firmly exclude a role for CTSL-mediated C3 activation in Th2 cell biology requires more extensive studies.

CTSL-mediated intrinsic C3 activation is sufficient to provide the autocrine complement receptor signals required for human effector cytokine induction because IFN-γ production is unaltered in serum-free media or in the presence of the C3 convertase inhibitor Compstatin (which readily prevents C3 convertase assembly on guinea pig red blood cells) (Figures S4D–S4F).

In mice, we found that resting CD4^+^ T cells also expressed C3 and CTSL and that mouse CTSL also processed mouse C3 into C3a and C3b (Figures S4G and S4H). As expected, CD4^+^ T cells from *C3*^–/–^ mice exhibited diminished Th1 cell-mediated responses under in vitro Th1 cell-skewing conditions (Figure S4I). However, T cells from *Ctsl*^*–/–*^ mice in which CTSL expression was only maintained in the thymic epithelium (to ensure normal T cell selection because CTSL is required for major histocompatibility complex class II molecule [MHC II] processing [Reiser et al., 2010]) unexpectedly had a normal, rather than reduced, Th1 cell phenotype (Figure S4J), indicating that murine CD4^+^ T cells process C3 into C3a in a CTSL-independent manner.

### In Vivo Generated Auto-Reactive T Cells Can Be “Normalized” by CTSL Inhibition

**Figure 5:**
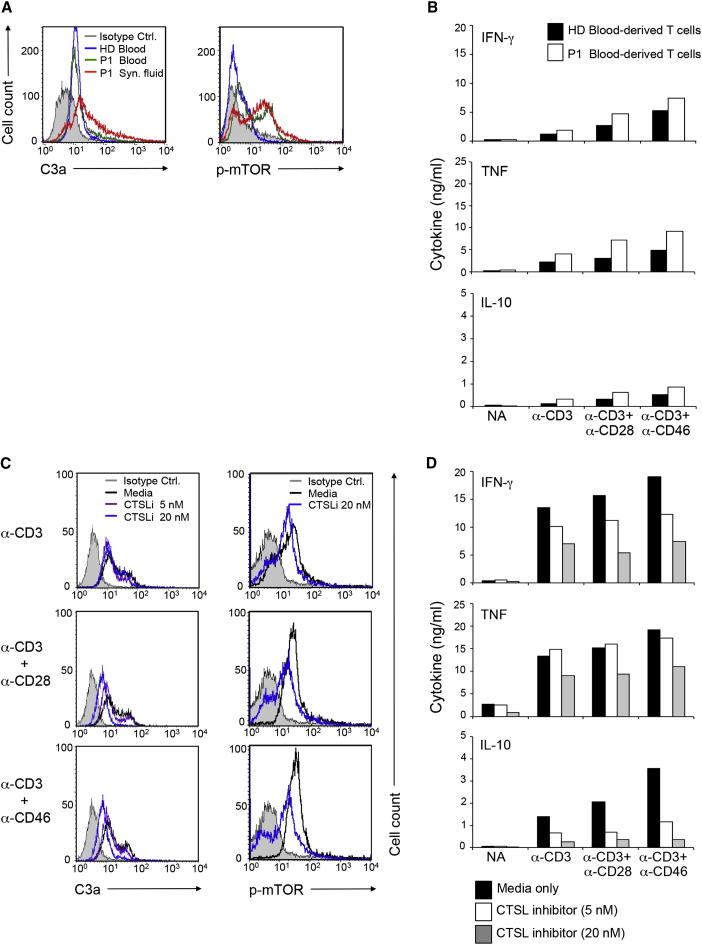
Enhanced Cytokine Production by T Cells in the Synovial Fluid from a Patient with Juvenile Arthritis Is Normalized by CTSL Inhibition (A) CD4^+^ T cells from blood of a healthy donor (HD) or blood and synovial fluid (Syn. fluid) of a patient with juvenile arthritis (JA) were assessed for intracellular C3a and mTOR immediately after purification. (B) Cytokine production of the stimulated HD and JA P1 peripheral blood T cells were assessed at 18 hr postactivation. (C) Intracellular C3a and activated mTOR amounts and (D) cytokine production of stimulated T cells from synovial fluid of JA P1 in presence of shown CTSL inhibitor (CTSLi) concentrations assessed at 18 hr. The addition of 5 nM CTSLi had no effect on cell viability. Supplementation of media with 20 nM CTSLi had no effect on nonactivated and CD3-activated cells and reduced cell viability by 5% (±2.7%) in CD3 + CD28 and CD3 + CD46-activated cells (not shown). Data represent mean values of conditions performed in duplicate. See also Figure S5.

Autoreactive effector T cells producing increased amounts of IFN-γ in the inflamed synovial fluid of juvenile idiopathic arthritis (JA) patients have protein kinase B (PKB) hyperactivation rendering these cells resistant to suppression by regulatory T cells (Tregs) (Wehrens et al., 2011). Because C3aR induces PKB activation (Strainic et al., 2008) and PKB activation is required for mTOR function (Verbist et al., 2012), we explored whether the uncontrolled T cell induction at the inflamed sites of these patients is due to deregulation of CTSL-mediated C3 activation. Indeed, synovial fluid CD4^+^ T cells from a JA patient had increased intracellular C3a and heightened mTOR activation compared to peripheral blood T cells of this patient or a healthy donor (Figure 5A). The increased production of IFN-γ (as well as the CD46-mediated coinduction of IL-10 in Th1 cells [Cardone et al., 2010]) and TNF by synovial fluid T cells (compared to T cells from the blood, Figure 5B) could be “normalized” in a dose-dependent manner by addition of the CTSL inhibitor to the cultures (Figure 5C). Furthermore, CTSL inhibition was accompanied by dose-dependent reduction in intracellular C3a and mTOR activity (Figure 5D). Comparable data were obtained with peripheral blood T cells of four different patients with rheumatoid arthritis (RA) (Figures S5A and S5B).

These data demonstrate that CTSL inhibition “normalizes” the increased production of proinflammatory cytokines by T cells from patients with autoimmune arthritis. Our finding that this is accompanied by a CTSL inhibitor dose-dependent reduction in intracellular C3a and mTOR activity suggests that the effect is, at least in part, mediated by decreased intracellular C3 activation.

### Natural Regulatory T Cells Engage Distinct Complement Receptor Pathways

**Figure 6:**
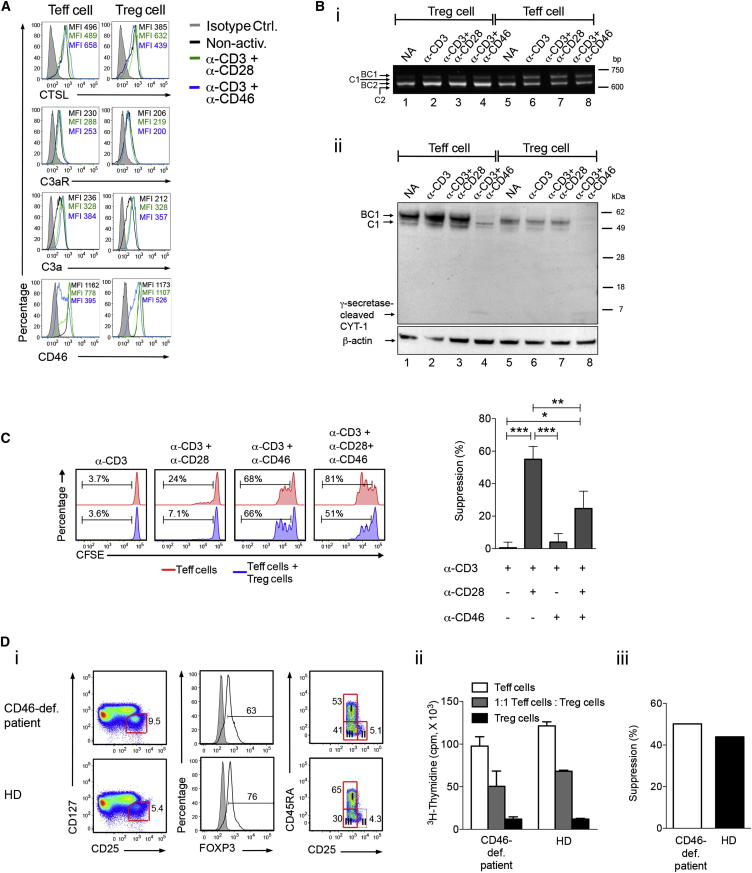
Effector and Regulatory T Cells Engage Distinct Complement Receptor Pathways (A) CTSL and complement protein expression in regulatory (Treg) and effector (Teff) T cells in resting and activated (1 hr) states. (B) CD46 mRNA (i) and protein expression (ii) in resting and activated (12 hr) Treg and Teff cells. (C) Effect of CD46 activation on Treg cell suppressive activity at a 1:1 Treg:Teff cell ratio. Results shown in (A)–(C) are derived from three independent experiments (n = 3) with the mean ± SD (C). BC1, C1, BC2, and C2 refer to the proteins forms and differently spliced mRNAs coding for the four protein isoforms of CD46. The mRNAs for C1 and C2 in (Bi) and the γ-secretase-processed CYT-1 of CD46 in (Bii) give weak signals at this exposure time but are clearly visible upon overexposure (data not shown). (D) Phenotype of Treg cells from a CD46-deficient (CD46-def.) patient and age- and sex-matched healthy controls (HD). Shown are (i) percentage of bulk CD4^+^CD25^hi^CD127^lo^ Treg cells, FOXP3 expression and percentages of Treg cell subpopulations I (CD4^+^CD25^hi^CD127^lo^CD45RA^+^), III (CD4^+^CD25^hi^CD127^lo^CD45RA^−^), and II (CD4^+^CD25^bright^CD127^lo^CD45RA^+^), (ii) suppressive function of Treg cells via ^3^H-thymidine incorporation measurement in 1:1 coculture, and (iii) calculated percentage suppression with mean values ± SD. ^∗^p < 0.05; ^∗∗^p < 0.01; ^∗∗∗^p < 0.005. See also Figure S6.

By using T cells isolated from arthritis patients, we demonstrated that increased CTSL-mediated activation of C3 is connected with uncontrolled pathological effector T cell function. We next explored the reverse situation and assessed the “CTSL-C3 system” in natural regulatory T (Treg) cells (Kitagawa et al., 2013) as local complement activation strongly impacts on the generation of these cells (Yamaguchi et al., 2011)—notably, blockage of C3aR and C5aR signaling on mouse and human naive CD4^+^ T cells leads to default induction of suppressive TGF-β-producing FOXP3^+^ T cells (Strainic et al., 2013). Correspondingly, C3aR and C5aR engagement on mouse CD4^+^CD25^hi^Foxp3^+^ natural Treg cells diminishes their suppressive activity (Kwan et al., 2013). We reasoned that human Treg cell induction or function could involve differences in intracellular complement activation or receptor signaling pathways. Initial assessment of CTSL, C3aR, C3a, and CD46 revealed no substantial differences in the profiles of these molecules between effector T cells and Treg cells in the resting or activated states (Figure 6A). Treg cells demonstrated comparable CD46 downregulation, which is observed upon CD46 crosslinking (Le Friec et al., 2012), at 2 hr postactivation (data not shown). However, Treg cells failed to increase expression of CD46 isoforms bearing the cytoplasmic tail 1 (CYT-1) upon activation (Figure 6B). CD46 is commonly expressed in four distinct isoforms. These arise from alternative mRNA splicing of regions coding for differently glycosylated extracellular domains (“BC” and “C”) and two distinct cytoplasmic tails, CYT-1 and CYT-2, generating BC1, C1, BC2, and C2 isoforms (Liszewski et al., 1991; 2005). We have previously shown that CYT-1 is required for IFN-γ production in CD4^+^ T cells (Cardone et al., 2010), whereas CYT-2 mediates contraction of IFN-γ production (Ni Choileain et al., 2011). The notion that Treg cells might have “disabled” the CD46-CYT-1 pathway required for Th1 cell induction is supported by our observation that CD46 activation did not abrogate suppressive capacity of Treg cells (Figure 6C; note that the apparent reduced suppression observed in the presence of CD46 antibodies is due to substantially increased Teff proliferation after CD46 stimulation [Kemper et al., 2003]) and does not induce IFN-γ or IL-10 in these cells (Grossman et al., 2004). Additionally, CD4^+^ T cells from a rare CD46-deficient patient that lacks productive Th1 cell-mediated responses (Le Friec et al., 2012) contained normal numbers of Treg cells (total or subpopulations I, II, and III [Miyara et al., 2009]), and these cells retained suppressive capacity (Figure 6D). These data suggest that, although Treg cells express all complement components required for proinflammatory cytokine production and generate intracellular C3a, it is the CD46 CYT-1 signaling that is particularly disengaged in Treg cells, enabling this T cell population to remain in an “anti-inflammatory state” (Figure S6).

### Intracellular C3 Activation Is Ubiquitous in Human Cells

**Figure 7:**
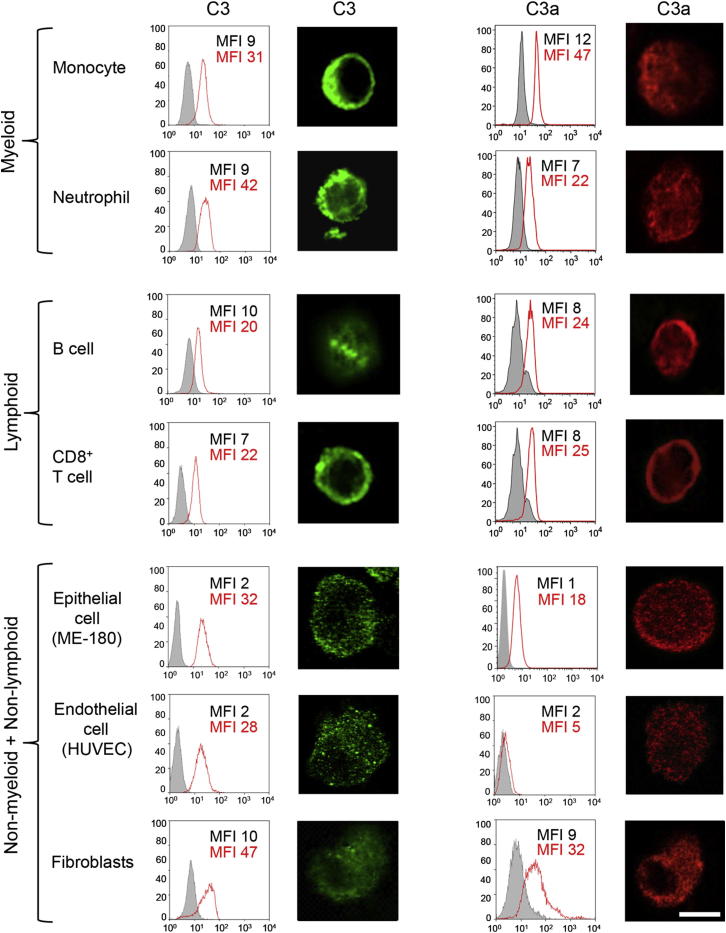
Intracellular C3 Stores and “Tonic” Intracellular C3a Generation Occurs in Myeloid, Lymphoid, and Nonmyeloid, Nonlymphoid Cell Populations Freshly isolated monocytes, neutrophils, CD8^+^ T cells, B cells, and cultured epithelial cells, endothelial cells, and fibroblasts were assessed for presence of intracellular C3b by flow cytometry and confocal microscopy image analyses (first and second column of panels, respectively), as well as for C3a (third and fourth column of panels, respectively) in the resting state. Results shown are representative of three independently performed experiments (n = 3). Scale bar represents 10 μM. MFI, mean fluorescence intensity.

To determine whether intracellular C3 activation is T cell-specific or not, we analyzed cells of myeloid (monocytes and neutrophils), of lymphoid (CD8^+^ T cells and B cells), and of nonmyeloid, nonlymphoid (epithelial cells, endothelial cells, and fibroblasts) origin and found that all of these cells not only contain stores of C3 in the resting state (Figure 7, two left panel columns) but also generate “tonic” intracellular C3a (Figure 7, two right panel columns). Thus, it appears that intracellular C3 activation might be a general phenomenon and not a T cell-restricted occurrence.

Overall, our results indicate an intracellular pathway of C3 activation that in T cells is catalyzed by CTSL and occupies a central niche in basal cellular homeostasis and in immunological T cell effector function during infection.

## Discussion

An emerging paradigm is that immune cell-derived complement activation fragments, as opposed to serum-circulating complement components, are the key players in initiating and regulating effector and regulatory T cell responses (Heeger and Kemper, 2012). The exact mechanisms by which CD4^+^ T cells generate C3a and C3b rapidly upon TCR stimulation are ill-defined. Studies performed in mice suggest that TCR activation induces gene transcription and protein secretion of C3 and factors required for C3 convertase assembly (factors B and D) by T cells and antigen-presenting cells (APC). This process subsequently leads to local C3 activation in the vicinity or the surface of these cells and engagement of complement receptors on T cells (Lalli et al., 2008; Liu et al., 2005; Strainic et al., 2008). Here, we present results suggesting that local complement activation by human CD4^+^ T cells follows a distinct mechanism and is, at least initially, C3 convertase-independent and occurs within the cell.

We demonstrate here that T cell-expressed CTSL cleaves C3 into active C3a and C3b fragments and thereby mediates the rapid local production of these fragments observed upon TCR activation. Unexpectedly, we found that TCR activation does not lead to induction of these proteins but that resting T cells contain intracellular pools of CTSL and C3. Furthermore, C3 activation by CTSL occurs continuously in the cell’s interior. We propose that this “tonic” intracellular C3a generation engages the C3aR, which, in resting cells, is expressed in large parts on lysosomes, but not on the cell surface, and by this mechanism sustains basal mTOR activation, required for homeostatic cell survival (André and Cota, 2012). Upon danger sensing (TCR activation), this “loaded” intracellular complement activation and receptor system is shuttled to the cell surface in effector cells to promote protective Th1 cell-mediated responses via C3aR and CD46-CYT-1-mediated signals (Le Friec et al., 2012). We suggest that C3aR-mediated signals induced from the cell surface differ from those triggered by intracellular C3aR activation, as has recently been shown in GPCR signaling (Irannejad et al., 2013). Interestingly, this C3aR and CD46-driven pathway is not operative in Treg cells because these cells are unable to upregulate CD46-CYT-1 expression. The lack of CD46 expression on somatic cells in mice (Tsujimura et al., 1998) indicates substantial differences between mice and humans in the signaling pathways used by their cells to integrate local complement activation. This notion is supported by results we obtained when we examined mouse T cells for this “CTSL-C3” axis: mouse cells also required processing of intracellular C3 for Th1 cell-mediated responses, but its cleavage was independent of CTSL. Of note, human CTSL is distinct from mouse CTSL in phylogeny, biochemical properties, and expression patterns (Reiser et al., 2010). Thus, although mouse CTSL processes mouse C3 in vitro, mouse CD4^+^ T cells seem to generate sufficient local C3a and C3b for Th1 cell induction in a CTSL-independent fashion, in line with the requirement for local extracellular C3 convertase assembly in this species (Strainic et al., 2008).

The most unexpected but likely most noteworthy outcome of our studies is the discovery of intracellular C3 processing. This represents a paradigm shift in our thinking about complement biology, demonstrating that complement activation is not only confined to plasma or hemolymph or the surface of cells but also occurs within the inside of cells. Importantly, this is not a T cell-specific phenomenon and, thus, intracellular complement activation is likely of broad physiological significance. From an evolutionary perspective, this makes sense because the early complement system was designed for activation of individual cells in order to prepare them for battle with injury, stress, or pathogens; and only later, when organisms began to develop organs, did complement components become secreted into the plasma or the hemolymph system (Le Friec and Kemper, 2009). The discovery of ubiquitous intracellular complement activation now advocates exploring additional roles for complement in other basic processes of the cell, e.g., gene translation and cell-cycle progression. Furthermore, crosstalk between complement and “classic” intracellular danger sensors such as the nucleotide-binding oligomerization domain (NOD)-like receptor (Chen et al., 2009) and the retinoic acid inducible gene-I (RIG-I)-like receptor systems (Thompson and Locarnini, 2007), previously dismissed because of the spatial separation of these systems, is now imaginable.

Our observations could also have implications for the design of next generation therapeutics-targeting complement. Interior pools of complement proteins and distinct modes of intracellular complement activation will need to be considered. T cells from patients with autoimmune arthritis displayed hyperactive intracellular complement activation and “correction” of this system by CTSL inhibition induced normalized Th1 cell-mediated responses. Thus, this intracellular pathway is amenable to therapeutic intervention with cell-permeable reagents. However, although our data indicate that the activation of C3 by CTSL plays a key role in T cell survival and function, CTSL may have additional, nonidentified substrates (Reiser et al., 2010) in human CD4^+^ T cells. We therefore cannot exclude that the CSTL-mediated induction of effector responses also involves the cleavage of other mediators or that blockage of these mediators by CTSL inhibition, in addition to prevention of C3 cleavage, in patients with arthritis contributes to the observed cytokine reduction. These possibilities should be explored.

Our data indicate that the C3aR is nonredundant for human Th1 cell effector function, whereas in mice the C5aR can partially substitute for Th1 cell induction in *C3ar*^–/–^ animals (Strainic et al., 2008), suggesting additional species-specific differences in the contribution(s) of complement to effector T cell induction. These differences extend also to pathways modulating Treg cell responses: In mice, lack of C3aR and C5aR engagement on naive CD4^+^ T cells, proposed to occur during interaction with tolerogenic antigen-presenting cells, fosters iTreg cell induction (Strainic et al., 2013). We suggest a different scenario in humans: intracellular complement activation and C3aR-engagement occurs in Treg cells but these cells do not upregulate the “proinflammatory” tail, CYT-1, of CD46 upon stimulation. Interestingly, highly inflammatory settings can alter CD46-mediated signals on human Treg cells and inhibit their function (Le Buanec et al., 2011). Thus, delineating what stimuli and pathways regulate CD46 isoform expression might have implications for therapeutic or environmental manipulation of Treg cell function.

Lastly, potential universal intracellular C3 activation suggests that a deregulation of this system could contribute to other diseases beyond Th1 cell-mediated autoimmunity, including cancer or allergies (Laplante and Sabatini, 2012). The C3-activating protease, however, is likely cell-specific; although we found that CTSL also processes C3 in monocytes (data not shown), C3 activation in lung epithelial cells, for example, is not mediated by CTSL. Therefore, identifying the C3-activating proteases within other immune-competent cells and understanding how intracellular complement activation is positively or negatively regulated are among important questions that now need to be addressed.

## Experimental Procedures

### Healthy Donors and Patients

Blood samples were obtained with appropriate ethical and institutional approvals (Wandsworth Research Ethics Committee, REC number 09/H0803/154). T cells were purified from buffy coats (NHSBT, Tooting, UK) or blood samples from healthy volunteers after informed consent. C3-deficient Patient 1 from France is 8 years old, with undetectable serum C3 and recurrent pyogenic infections from the age of 2 years on (Ghannam et al., 2008). Genomic sequencing of the *C3* gene of Patient 1 demonstrated a single nucleotide insertion at position 1648 in the maternal allele and a change from c.1648T to C (p.Ser^550^Pro) in the paternal allele. Patients 2 and 3 were 9.5- and 8-year-old siblings, respectively, with no detectable serum C3 (the *C3* gene mutations in these patients have not yet been defined; SM, personal communication), the elder having severe recurrent infections from an early age (Pekkarinen et al., 2013). Both patients take prophylactic amoxicillin. All patients were infection-free at the time of blood sampling; blood collection and processing was conducted with approval of the respective local ethics committees. An adult CD46-deficient patient with described defective Th1 cell-mediated responses was recruited in France (Couzi et al., 2008). This patient has hemolytic uremic syndrome (HUS) and common variable immunodeficiency (CVID), receiving monthly intravenous immunoglobulin. The patient had neither infection nor active HUS at the time of blood sampling. Adult patients with inflammatory arthritis (including rheumatoid arthritis and juvenile arthritis) were recruited. All patients had active disease with disease activity scores for 28 joints (DAS28) > 4.9, representing moderately severe activity, despite therapy with disease modifying antirheumatic drug methotrexate. Synovial fluid was obtained during therapeutic knee arthrocentesis.

### Antibodies, Proteins, and Inhibitors

Details are included in Supplemental Experimental Procedures.

### Enzymatic Reactions and Detection of C3 Cleavage Fragments

Recombinant CTSL was activated according to manufacturer’s protocol. Unless otherwise indicated, 150 ng of purified human C3 was incubated with 250 ng of CTSL in 100 μl PBS for different time points (unless otherwise indicated for 60 min) at 37°C ± addition of CTSL inhibitor (40 nM) or antibodies to CTSL that block or do not block C3 cleavage. Reaction mixtures were analyzed for C3 fragments by immunoblotting or using SilverXpress Silver Staining Kit (Life Technologies). C3 and CTSL cleavage reactions were performed at pH 5.6 (to mimic lysosomal CTSL activity) and pH 7.3 (to mimic secreted CTSL activity at alkaline pH) and yielded comparable results (data not shown). Enzymatic reactions for C3 and CTSG and CTSB, for C4 and C5 and CTSL, and for mouse C3 and mouse CTSL were performed similarly as noted in the text.

### T Cell Isolation, Activation, and Assessment of Apoptosis and Viability

T cell isolation and activation and measurement of viability or apoptosis were carried out with standard techniques (Supplemental Experimental Procedures).

### Other Cells

ME-180 and HUVEC, an epithelial cell line and endothelial cell line, respectively, from the American Type Tissue Culture Collection (ATCC; Manassas, VA), were grown in McCoys 5a media (Invitrogen), and EBM-2 (Lonza) both supplemented with heat-inactivated fetal calf serum (FCS). Human fibroblasts (ATCC) were cultured in Fibroblast Basal Medium (ATCC) according to manufacturer’s protocol. Human CD8^+^ T cells, monocytes, and B cells were identified within PBMC preparations by fluorochrome-conjugated antibodies to CD8, CD14, and CD19, respectively. Neutrophils were prepared from erythrocyte fractions of whole-blood Ficoll separations (Cardone et al., 2010) by hypotonic lysis.

### Confocal Microscopy and Colocalization Analysis

Fixed, permeabilized cells were stained with primary antibodies overnight at 4°C. Where indicated, staining with secondary antibodies was performed for 2 hr at room temperature. Cells were mounted with Fluoromount-G (SouthernBiotech, Birmingham, AL) and images were obtained in the KCL Nikon Imaging Centre by confocal fluorescence microscopy with either A1R SI Confocal Microscope (×60 objective) or Super Resolution Confocal Microscope (N-SIM) (×100 objective), both from Nikon™ (Surrey, UK). Pearson’s Correlation Coefficient was calculated with NIS Elements software version 4.03 (Nikon). At least ten layers in 3D plane were scanned for each sample and for all samples a cropped image of a minimum of 12 cells was used to determine a total of 5 colocalization coefficients. Median values for all layers and cells were calculated and used to plot Pearson’s Correlation Coefficient. Experiments were performed at least three times with three different healthy donors each time.

### Cytokine Measurements

Cytokines in cell supernatants were measured using human or mouse Th1 and Th2 or Th1, Th2 and Th17 Cell Cytometric Bead Arrays (BD), or by intracellular staining.

### RT-PCR and RNA Silencing

Details of all primers used and RNA silencing with siRNA are given in Supplemental Experimental Procedures.

### Statistical Analysis

Analyses were performed on GraphPad Prism (La Jolla, CA). Data are presented as mean ± SD or median (interquartile range, IQR) for parametric and nonparametric data, respectively, and compared with paired t tests with Bonferroni correction for multiple comparisons, Wilcoxon signed rank tests, the two-tailed Mann-Whitney test, or one-way ANOVA with a Tukey multiple comparison post hoc test, as appropriate. p values < 0.05 denoted statistical significance throughout.

## Supplemental Information

Document S1. Figures S1–S6 and Supplemental Experimental Procedures
