# Supplementary material for: Intracellular Complement Activation Sustains T Cell Homeostasis and Mediates Effector Differentiation
Source: Immunity. 2013 Dec 12;39(6):1143–57. doi: 10.1016/j.immuni.2013.10.018 (PMC3865363; doi:10.1016/j.immuni.2013.10.018)
Supplement: Document S1. Figures S1–S6 and Supplemental Experimental Procedures [file mmc1.pdf]

Immunity, Volume *39*

Supplemental Information

Intracellular Complement Activation Sustains T Cell Homeostasis and Mediates Effector Differentiation

M. Kathryn Liszewski, Martin Kolev, Gaelle Le Friec, Marilyn Leung, Paula G. Bertram, Antonella F. Fara, Marta Subias, Matthew C. Pickering, Christian Drouet, Seppo Meri, T. Petteri Arstila, Pirkka T. Pekkarinen, Margaret Ma, Andrew Cope, Thomas Reinheckel, Santiago Rodriguez de Cordoba, Behdad Afzali, John P. Atkinson, and Claudia Kemper

Supplemental Inventory

1. Supplemental Figures and Tables

Figure S1, related to Figure 1

Figure S2, related to Figure 2

Figure S3, related to Figure 3

Figure S4, related to Figure 4

Figure S5, related to Figure 5

Figure S6, related to Figure 6

2. Supplemental Experimental Procedures

3. Supplemental References

SUPPLEMENTAL FIGURES

FIGURE S1

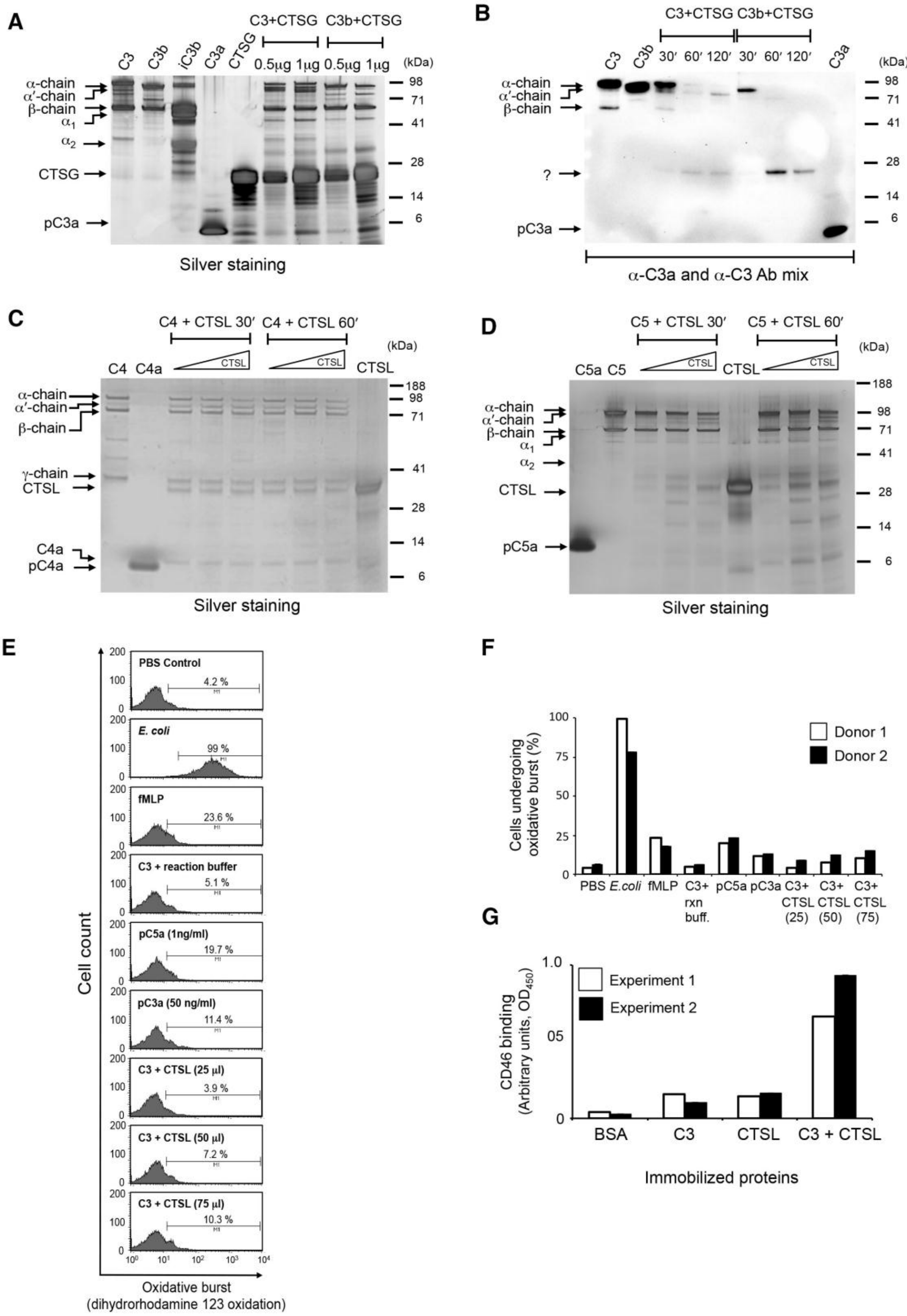

Figure S1. C3 is specifically cleaved by Cathepsin L and Cathepsin L-generated C3a and C3b are biologically active. (A and B) Cathepsin G (CTSG) does not cleave C3 into C3b and C3a. Purified C3 (150 ng) was incubated with activated recombinant human CTSG (0.5 or 1 μg) for 120 min (**A**) or with 0.5 mg for 30, 60 or 120 min (**B**) and reaction mixtures analysed by silver staining (**A**) or by Western blotting (**B**) for C3 cleavage fragment generation. For Western blot analysis, a mixture of antibodies recognizing C3, C3b and C3d and the C3a neo-epitope was used. Purified C3, C3b, and C3a (and iC3b for (**A**)) and recombinant CTSG were loaded as controls. Data shown are representative of three (*n* = 3) (**A**) and two (*n* = 2) (**B**) independently performed experiments. (**C and D**) Cathepsin L (CTSL) cleaves C4 into C4b and C4a-like fragments (**C**) but does not process C5 into C5a and C5b (**D**). Purified C4 or C5 (200 ng) was incubated with increasing amounts of activated recombinant human CTSL (250, 500 and 750 ng) for either 30 or 60 min and reaction mixtures analysed by silver staining for C4 (**C**) or C5 (**D**) cleavage fragment generation. Purified C4, C5, C4a, C5a, and recombinant CTSL alone were loaded as controls. Shown is one representative of two similarly performed experiments (*n* = 2). (**E**) CTSL-generated C3a induces oxidative burst in neutrophils. C3 (500 ng/150 μl reaction mixture) was incubated with CTSL (750 ng/150 μl reaction mixture) for 60 min. 25, 50 or 75 μl of the reaction mixtures were then used in the Phagoburst™ Assay according to the manufacturer's protocol. Oxidative burst was monitored by FACS analysis of fluorogenic dihydrorhodamine (DHR) 123 substrate-conversion. Positive controls included serum-opsonised *E. coli* as strong stimulus, fMLP (the chemotactic peptide N-formyl MetLeuPhe) and purified C5a as intermediate stimuli and purified C3a as weak stimulus. As negative controls, neutrophils were incubated with either PBS or with 75 μl of C3 treated with the CTSL reaction buffer only (no CTSL addition). Shown in (**E)** is one experiment of two similarly performed and in (**F)** the summary of data derived with cells isolated from two different donors and the means of conditions performed in triplicate (*n* = 2). (**G**) Reaction mixtures (75 μl) and controls were also immobilized onto ELISA plates, incubated with sCD46 (100 ng) and CD46 binding assessed with TRA-2-10 anti-CD46 antibody. Shown are results (mean of conditions performed in duplicate) from two separate experiments (*n* = 2). Figure S1, related to Figure 1.

Figure S2

Supplementary Table 1. C3a levels in T cells activated in the presence or absence of cathepsin L blockage

	INTRACELLULAR					EXTRACELLULAR				
	**Isotype ctrl.**	**Media only**	**CSL Inh.**	**α-CSL blocking**	**α-CSL non-block.**	**Isotype ctrl.**	**Media only**	**CSL Inh.**	**α-CSL blocking**	**α-CSL non-block.**
**NA**	*9.5 (± 2.1)	14.7 (± 1.0)	11.3 (± 1.0)	nd	nd	12.3 (± 1.0)	9.8 (± 2.4)	10.2 (± 1.9)	nd	nd
**α-CD3**	6.9 (± 2.4)	16.3 (± 1.1)	11.2 (± 1.7)	16.9 (± 1.6)	17.9 (± 2.0)	13.7 (± 1.3)	42.9 (± 4.6)	35.2 (± 4.1)	28.4 (± 2.7)	58.3 (± 6.2)
**α-CD3 + α-CD46**	5.3 (± 2.8)	16.5 (± 1.2)	9.8 (± 1.0)	15.01 (± 1.3)	19.0 (± 1.2)	10.7 (± 1.0)	60.2 (± 8.7)	33.4 (± 5.5)	42.0 (± 6.8)	64.9 (± 8.4)

Flow cytometric analysis for intracellular and extracellular C3a presence in non-activated $CD4^+$ T cells and T cells activated with immobilized Abs to CD3 or CD3 and CD46 for 1 h. CSL inh., cathepsin L inhibitor; non-block., non-blocking Ab to CSL; nd, not determined. Data are derived from three independently performed experiments using a different donor each time. *, data are represented as mean fluorescence intensity (MFI) with MFI deviations in parentheses.

A

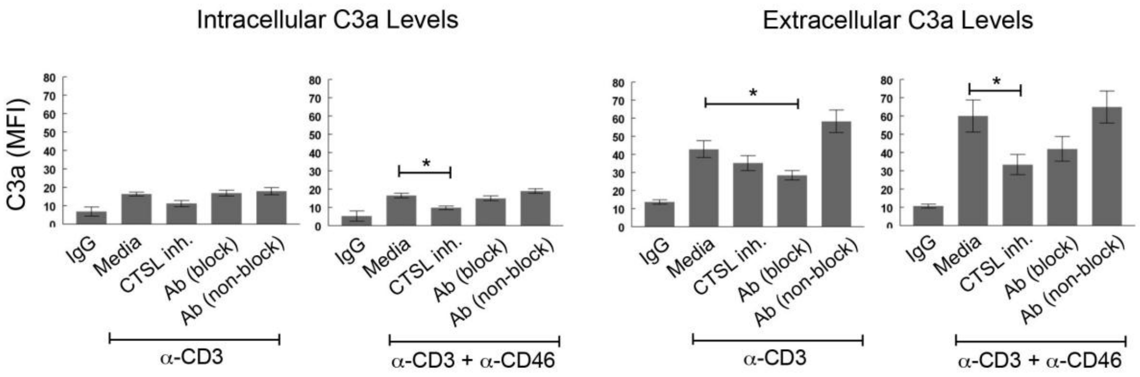

B

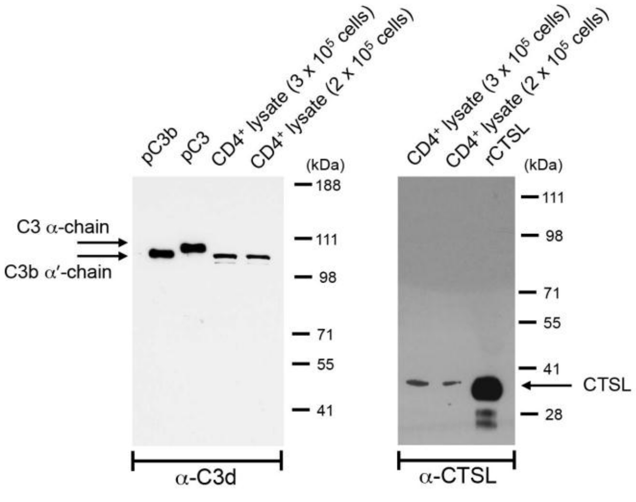

Figure S2. Cathepsin L inhibition reduces intracellular and extracellular C3a generation in activated human CD4^{+} T cells and resting CD4^{+} T cells contain mostly the processed α′-chain of C3. (**A**) Graphical representation of the data in **Table S1**. IgG, wells coated with isotype-specific IgGs; Media, non-activated cells incubated with media alone; CTSL-Inh., cathepsin L inhibitor; Ab (block), function blocking mAb to CTSL; Ab (non-block), mAb to CTSL that does not block its activity. Data represent the mean values ± SD of mean fluorescence intensity (MFI) and are derived from three independent experiments (n = 3). *, $p < 0.05$ when comparing intracellular C3a levels of CD3 and CD46-activated cells with and without inhibitor or when comparing extracellular C3a levels between CD3-activated or CD3 and CD46-activated cells with and without inhibitor (paired t-test with Bonferroni correction). (**B**) Freshly purified human CD4^{+} T cells were lysed. Cell lysates were separated using polyacrylamide gel electrophoresis (10% gel) and then analysed by Western blotting using a rabbit antibody against human C3d (left panel) or a rabbit antibody raised against human cathepsin L (CTSL). Human purified C3b (pC3b) and C3 (pC3) and recombinant human CTSL (rCTSL) were loaded as controls. Data shown are representative of four independently performed experiments (n = 4). Figure S2, related to Figure 2.

FIGURE S3

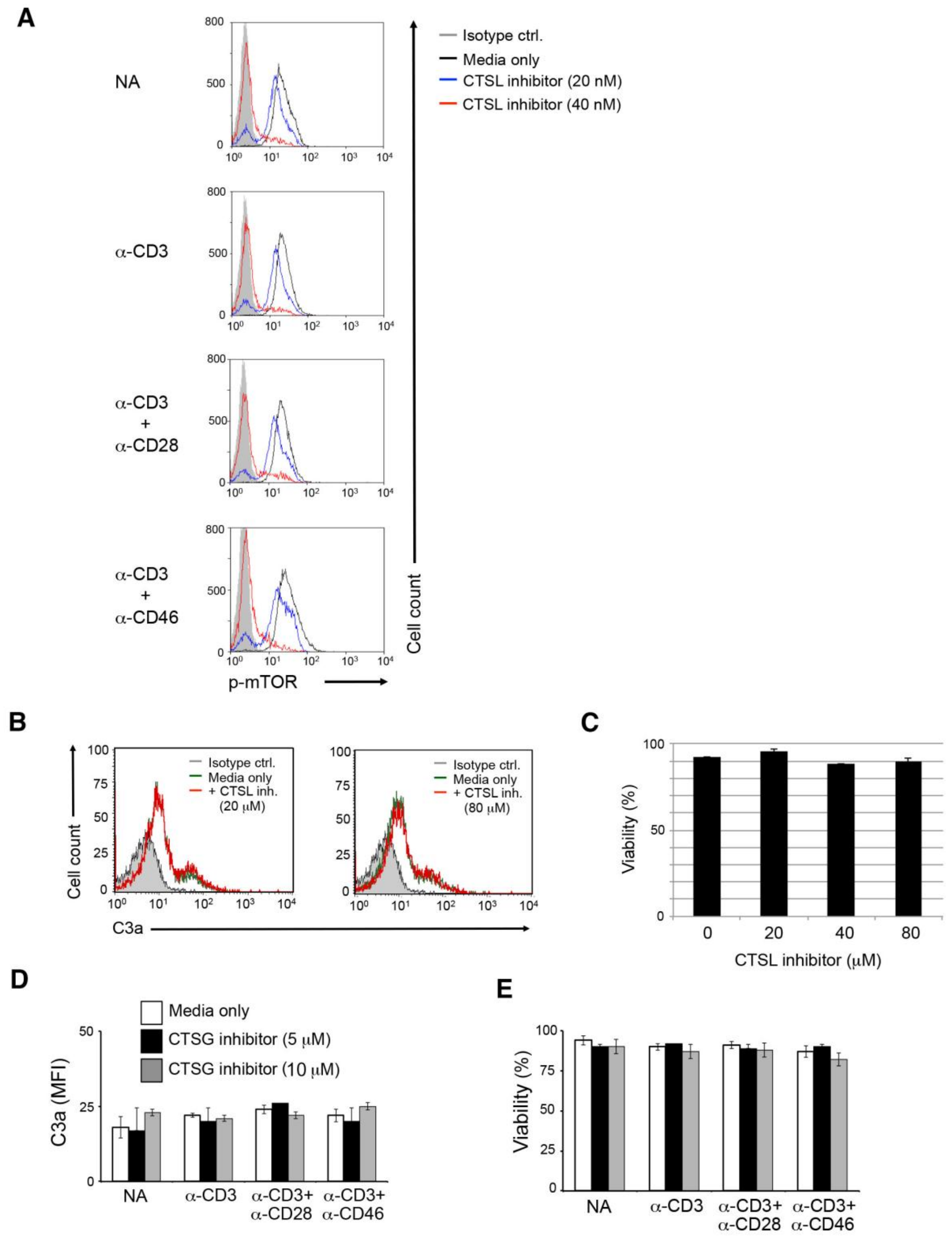

Figure S3. Cathepsin L inhibition reduces mTOR activity in activated T cells but does not affect C3a generation or viability in lung epithelial cell. (**A**) Freshly purified CD4^{+} T cells were either left non-activated (NA) or activated with the depicted immobilised antibodies in the presence or absence of either 20 nm or 40 nM cathepsin L (CTSL) inhibitor. At 18 h post-activation, mTOR activity was

assessed by measuring phosphorylated mTOR (p-mTOR) by intracellular FACS analysis. The plots shown are representative of three independently performed experiments using T cells from three different donors each time (n = 3). (**B**) Cathepsin L (CTSL) inhibitor treatment does not alter C3a generation in lung epithelial cells. Human Bronchial Epithelial cells (16HBE14o) were seeded in 48-well plates at a concentration of 16 x 10^5 cells/ml and cultured to confluence. Cells were then treated with either 20 μM or 80 μM cathepsin L (CTSL) inhibitor for 12 h and intracellular C3a measured by FACS analysis. FACS plots are representative of three independently performed experiments (n = 3). (**C**) CTSL inhibitor treatment does not affect cell viability. Experiments were performed as under (**C)** but with the inclusion of a 40 μM CTSL inhibitor concentration and cell viability assessed by Annexin V and propidium iodide staining. Data are presented as mean ± SD and are derived from two independent experiments with each condition performed in duplicate (n = 2). (**D and E**) Cathepsin G inhibition does not affect C3a generation or cell viability of human $CD4^+$ T cells. Purified $CD4^+$ T cells were activated with depicted immobilised antibodies in media only, or in media with addition of 5 μM or 10 μM cathepsin G (CTSG) inhibitor. (**D**) C3a generation (in permeabilised and fixed cells) and (**E)** cell viability were measured 12 h post-activation. Data ± SD shown are derived from three independently performed experiments using a different donor each time (n = 3). Figure S3, related to Figure 3.

FIGURE S4

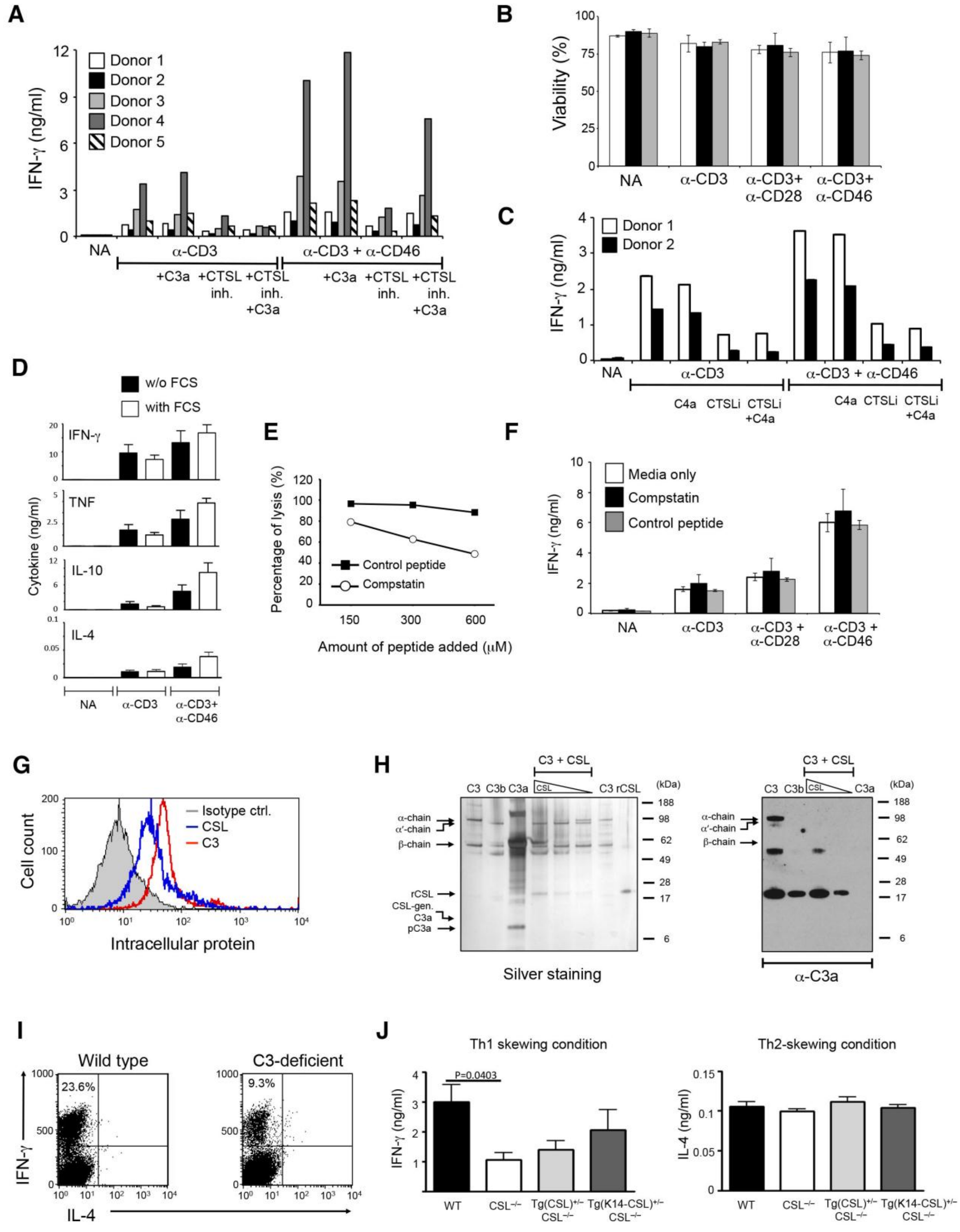

Figure S4. Th1 induction in human CD4$^+$ T cells depends on Casthepsin L-generated C3a and C3b whereas mouse CD4$^+$ T cells can generate C3a locally in a cathepsin L-independent fashion. (**A and B**) Th1 induction in human CD4$^+$ T cells is dependent on cathepsin L-generated C3a. (**A**)

Purified $CD4^+$ T cells were either left non-activated (NA) or were activated with the depicted immobilised antibodies in media only, in media with addition of 5 nM cathepsin L (CTSL) inhibitor (a concentration that does not reduce cell viability (**B**)) or in media with CTSL inhibitor and C3a (100 ng/ml). IFN-γ production by cells was measured 36 h post-activation. Data shown are the mean of activation conditions performed in duplicate derived from five donors. (**C**) Addition of C4a fails to rescue CTSL inhibition-mediated reduction in IFN-γ production. Experiments were performed as under (**A**) but with the addition of purified C4a (100 ng/ml) instead of C3a. Shown are results as mean of activation conditions performed in duplicate from three independent experiments using T cells from a different donor each time. (**D**) Th1 and Th2 induction in $CD4^+$ T cells is comparable in FCS-free and FCS-supplemented media. T cells were activated as depicted in media with or without heat-inactivated fetal calf serum (FCS) and culture supernatants assessed for cytokine production 36 h post-activation. Data shown are the mean ± SD of seven independently performed experiments (n = 7). (**E**) Compstatin inhibits C3 convertase surface formation and lysis of guinea pig erythrocytes exposed to 8% human serum in a dose-dependent manner but (**F**) addition of Compstatin (600 mM) did not alter IFN-γ production when added to T cells during activation. Data in (**E**) and (**F**) are derived from three independently performed experiments (n = 3). (**G**) Resting splenic mouse $CD4^+$ T cells express C3 and cathepsin L (CSL) as determined by intracellular FACS analysis. Shown is one representative staining of three performed using a different mouse each time (n = 3). (**H**) Mouse CSL cleaves mouse C3 into a C3b and C3a-like fragment. Mouse C3 (100 ng) was incubated with mouse CSL (250 ng) for 60 min and C3 fragment generation monitored by silver staining (left panel) or Western blotting (right panel). Note that the Ab used for Western blotting only recognizes C3a contained within the uncleaved α-chain. Shown is one representative of three similarly performed experiments (n = 3). (**I**) $CD4^+$ T cells from C3-deficient mice have reduced *in vitro* Th1 responses. $CD4^+$ T cells were isolated from the spleens of wild type control mice or C3-deficient mice and expanded under Th1-skewing conditions for 7 days. Cytokine production was assessed by intracellular staining after 4 h of PMA and ionomycin re-stimulation of cells. Data shown are representative of results derived from three animals in each group (n = 3). (**J**) Defective *in vitro* Th1 induction in T cells from CSL-deficient mice

is rescued by thymic CSL expression. T cells were isolated from the spleens of cathepsin L-deficient ($CSL^{-/-}$) or matching wild type (WT) mice or from $CSL^{-/-}$ mice transgenic for one allele of CSL $Tg(CSL)^{0/+}CSL^{-/-}$ or $CSL^{-/-}$ mice with conditional transgenic expression of CSL in the thymic epithelium ($Tg(K14\text{-}CSL)^{0/+}CSL^{-/-}$ and cultured an expanded under Th1- or Th2-skewing conditions for 7 days. IFN-γ and IL-4 production by these cells was assessed after 4 h PMA and ionomycin re-stimulation. Data represent the results derived from three animals in each group ± SD (n = 3). Statistical significance was determined using the two-tailed Mann-Whitney test. Figure S4, related to Figure 4.

FIGURE S5

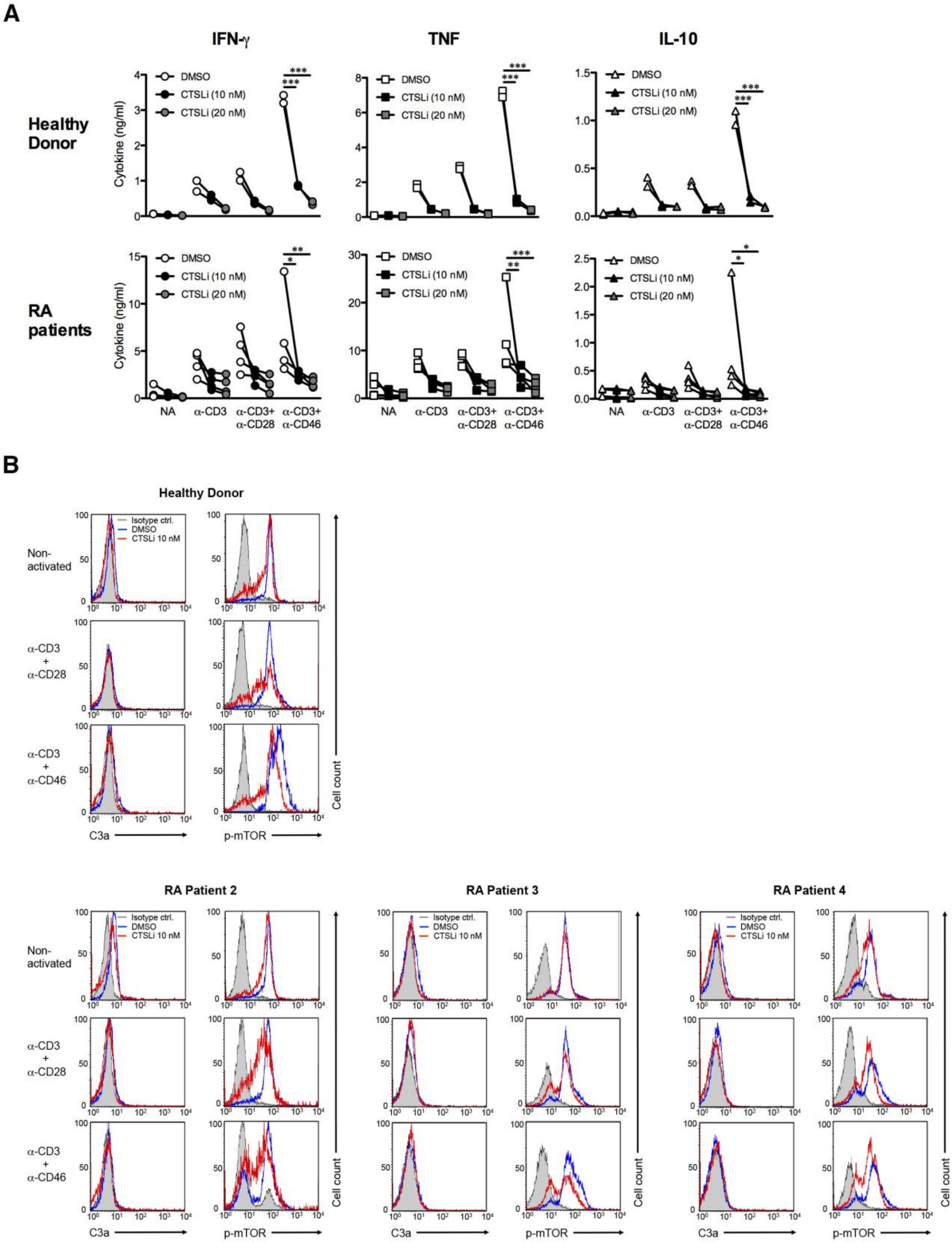

Figure S5. Increased cytokine production by circulating T cells from rheumatoid arthritis patients is normalized by inhibition of cathepsin L. (**A**) Cytokine production by previously frozen

CD4$^+$ T cells from a healthy donor (upper panels) and from four different rheumatoid arthritis (RA) patients (lower panels) at 18 h post activation with or without addition of 10 or 20 nM CTSL inhibitor (CTSLi) ($n = 4$). Frozen T cells from the same healthy donor were used as technical replicate in two separate experiments to control for potential effects on T cell phenotype during the freezing procedure and storage. Cytokine secretion was normalized for DMSO control and viable cells numbers (with no culture condition containing > 15 % dead cells). (**B**) Intracellular C3a levels and mTOR activation (measured as phosphorylated mTOR, p-mTOR) status 18 h post-activation from the healthy donor (shown is one representative FACS plot of two independently performed experiments) and RA Patients 2, 3 and 4. RA P1 data on C3a and mTOR are not available. *$p < 0.05$; **$p < 0.01$; ***$p < 0.005$ determined using one-way repeated measures ANOVA with Tukey *post-hoc* test. Figure S5, related to Figure 5.

FIGURE S6

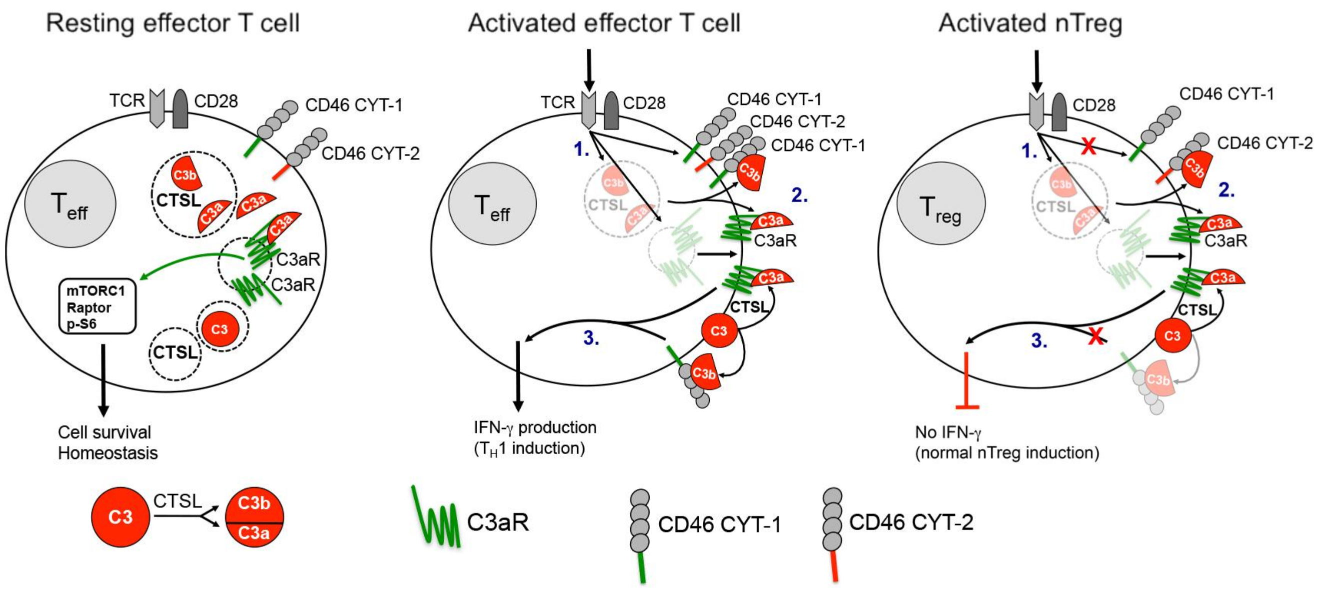

Figure S6. Model for the role of CTSL-generated C3a and C3b and their respective receptors, C3aR and CD46, in cell survival and induction of effector function in CD4$^+$ T cell sub-populations. Tonic CTSL-mediated generation of intracellular C3a supports homeostatic T cell survival via mTOR activation (left panel). T cell receptor activation induces translocation of intracellular stores of C3aR to the cell surface (1, middle panel), amplifies intracellular CTSL-mediated generation of C3a and C3b and induces extracellular, cell surface CTSL-mediated C3 activation. Extracellular C3aR and CD46 engagement by C3a and C3b (2, middle panel) subsequently leads to induction of T cell effector function (3, middle panel). In Tregs, Cyt-1-bearing CD46 isoforms are not upregulated upon TCR activation and IFN-γ production is not initiated (right panel). Figure S6, related to Figure 6.

SUPPLEMENTAL EXPERIMENTAL PROCEDURES

Mice. Previously described C3-deficient mice (Wessels et al., 1995) were provided with matching wild type C57BL/6 animals by Prof. M. Botto (Imperial College, London). *Ctsl*-deficient and transgenic Tg(K14-hCV);*Ctsl*-deficient mice were generated as previously described (Hagemann et al., 2004) and back crossed onto C57Bl/6 background for at least 8 generations. All animals were cared for in accordance with institutional guidelines.

Antibodies, proteins and inhibitors. Cell-stimulating mAbs were bought from BD Biosciences, San Diego, CA (anti-human CD28, CD28.2; anti-mouse CD3, 145-2C11; anti-mouse CD28, 37.51), purified from a specific hybridoma (anti-CD3; OKT-3) or generated in-house (anti-CD46; TRA-2-10 (Wang et al., 2000). Anti-mouse IL-4 (16-7041-81) and IFN-γ (16-7311-81) mAbs were from eBioscience (San Diego, CA). Anti-human CD25 (558142), CD8 (555635), CD14 (561707), CD19 (555415) and CD46 (clone E4.3) were obtained from BD; anti-CD127 (50-1278) from eBioscience and anti-CD45RA from Biolegend (Cambridge, UK; 304127). CD46 CYT-1 was detected using an antibody from Prof. Maggie So (University of Arizona, AZ (Weyand et al., 2006)). Human C3/C3b expression was analyzed with rabbit anti-C3d (ab17453) from Abcam (Cambridge, MA) and chicken anti-C3 (GW20073F) from Sigma Aldrich (Saint Louis, MO). The antibody detecting human C3a neo-epitope was a gift from Jörg Köhl (University of Lübeck, Germany (Hartmann et al., 1993)); mouse monoclonal antibody detecting C3a within the C3 α-chain was from Abcam (ab36385). C3aR antibodies for microscopy and FACS were from Abcam (ab126250) and BD Biosciences (561178). C3 cleavage-blocking rabbit antibody to human CTSL (ab58991; this antibody also detects mouse cathepsin L), non-blocking mouse antibody to human CTSL (ab6314) and rabbit polyclonal antibodies to calnexin (ab22595), EEA1 (ab2900), Lamp1

(ab24170), Rab5 (ab13253), and β-actin loading control (ab8226) were purchased from Abcam. Antibodies recognizing p-mTOR (2971S), p70 S6 Kinase (9205S), human RagC (3360) and the mTOR Regulation Antibody Sampler Kit (9864) were bought from Cell Signaling Technology (Danvers, MA). FOXP3 Staining Kit from bought from eBioscience. The APEX™ Biotin-XX Antibody Labeling Kit (A1045) from Life Technologies (Carlsbad, CA) was used to biotinylate antibodies and BD Cytofix/Cytoperm™ Fixation/Permeabilization Solution Kit (555028) used to fix/permeabilize cells. Recombinant Human cathepsin B (953-CY-010) and CTSL (952-CY-010) and mouse CTSL (1515-CY-010) were obtained from R&D (Minneapolis, MN). Recombinant human cathepsin G (CTSG-1649H) was bought from Creative BioMart (Shirley, NJ). Mouse IL-4 (BMS338) and IL-12 (14-8121) were obtained from eBioscience. Pertussis toxin from *Bordetella pertussis* was from Sigma Aldrich (P7208) and serum purified C3 (A113), C3b (A114), iC3b (A115), C3a (A118), C4 (A105), C4a (A106), C5 (A120) and C5a (A144) were from Complement Technologies Inc. (Tyler, TX). Mouse C3 was purified from mouse serum as previously described (Van den Berg et al., 1989). Cell-permeable inhibitors to CTSL (ALX-260-133-M001) and cathepsin G (CA-074 Me) were bought from Enzo Life Sciences. (Exeter, UK) and Compstatin (2585) and Compstatin control peptide (3765) purchased from Tocris Bioscience (Bristol, UK). Guinea pig blood cells in Alsever's solution (PB029) from TCSBiosciences (Buckingham, UK) were used in red blood cell lysis assays.

T cell isolation and activation. PBMCs were separated to $CD4^+CD25^-$ effector (Teff) cells and $CD4^+CD25^+$ regulatory cells (Tregs) using MACS human $CD4^+CD25^+$ Regulatory T cell Isolation Kit (Miltenyi Biotech) according to manufacturer's instructions. Purity of isolated lymphocyte fractions was typically >95%. Cell viability and/or apoptosis was measured using the Vybrant® Apoptosis Assay Kit (YO-PRO®-1/Propidium Iodide) (Molecular Probes).

Teffs were activated in 48-well culture plates (2.5–3.5 x 10^5 cells/well) coated with mAbs to CD3, CD28 ± CD46 (2.0 μg/ml each) and addition of 25 U/ml rhIL-2. Treg suppression assays were carried out as previously described using the CFSE method (Afzali et al., 2013). For suppression assays using CD46-def. Tregs, due to small cell numbers, incorporation of tritiated thymidine was used as readout, as described (Afzali et al., 2011). Mouse $CD4^+$ T cells, isolated from spleens using mCD4 Microbeads (Miltenyi), were activated in 48-well plates coated with antibodies to mouse CD3+CD28 in the presence of rIL-2 (20 ng/ml), mIL-12 (20 ng/ml) and anti-mIL-4 (10 μg/ml) for Th1-skewing or in the presence of rIL-2 (20 ng/ml), mIL-4 (20 ng/ml) and anti-mIFN-γ (10 μg/ml) for Th2-skewing for 3 days, then expanded in rIL-2 until day 7. Cytokine production was assessed after CD3 + CD28 re-stimulation over night or exposure to PMA and ionomycin for 4 hours.

RT-PCR. Primers used to quantify mRNA transcription shown in Figure 1C: *C3*, Forward (F) ^{5}CAA CAA GTT CGT GAC CGTGC^{3} and Reverse (R) ^{5}CCA GGG GTG TAG ATG GTC TTG^{3}; *CTSL*, (F) ^{5}ACG CCT TTG GAG ACA TGA CCC^{3} and (R) ^{5}TGG GGG CCT CAT AAA ACA GAG^{3}; *C3aR*, (F) ^{5}AAC CTG CTG ATG TGG TCT CAC^{3} and (R) ^{5}CGC TAG GGA ACA GCT TTA AAT GAG^{3}, *CD46 (all four isoforms),* (F) ^{5}GTG GTC AAA TGT CGA TTT CCA GTA GTC G^{3} and (R) ^{5}CAA GCC ACA TTG CAA TAT TAG CTA AGC CAC A^{3} (Wang et al., 2000). Primers used to detect specifically sequences coding for the *C3* β-chain, the C3a portion and the α-chain shown in Figure 3F: *C3 exon 2 and 3 (β-chain),* (F) ^{5}CAA GGG GAT GTT CCA GTC AC^{3} and (R) ^{5}ATG AAG AGG TAC CCG CTC TG^{3}; *C3 C3a portion*, (F) ^{5}CCT GGA CTG CTG CAA CTA CA^{3} and (R) ^{5}ACT CAC TTC GGG AAA CGA TG^{3}; *C3 α-chain,* (F) ^{5}AGC TCA CGG AGA AGC GAA T^{3} and (R) ^{5}CCA GGA AGA CCT TCT TGC AC^{3}; *ACTB (β-actin) control*, (F) ^{5}AGC ACA GAG CCT CGC CTT T^{3} and (R) ^{5}CAC GCA GCT CAT TGT AGA AG^{3}. PCR reactions were performed using the

QIAGEN OneStep RT-PCR Kit according to the manufacturer's protocol and with the specific annealing temperatures/times calculated for each primer pair.

RNA silencing. siRNA targeting human C3aR (sc-42840) and negative control siRNA (sc-37007) were purchased from Santa Cruz Biotechnology (Dallas, TX) and delivered into primary human $CD4^+$ T cells by electroporation (2 x 10^6 cells ml/transfection buffer (Ambion); 3 μg/ml siRNA; 200 V and 325 mF using the Bio-Rad Gene Pulser (Bio-Rad Laboratories, Hercules, CA). Transfection efficiency was consistently >80 %; protein knockdown (between 65-75% in all donors) peaked at 18-24 hours post-transfection.

SUPPLEMENTAL REFERENCES

Afzali, B., Mitchell, P.J., Scottà, C., Canavan, J., Edozie, F.C., Fazekasova, H., Lord, G.M., John, S., Barber, L.D., Hernandez-Fuentes, M.P., et al. (2011). Relative resistance of human CD4(+) memory T cells to suppression by CD4(+) CD25(+) regulatory T cells. Am J Transplant *11*, 1734–1742.

Afzali, B., Edozie, F.C., Fazekasova, H., Scotta, C., Mitchell, P.J., Canavan, J.B., Kordasti, S.Y., Chana, P.S., Ellis, R., Lord, G.M., et al. (2013). Comparison of regulatory T cells in hemodialysis patients and healthy controls: implications for cell therapy in transplantation. Clin J Am Soc Nephrol *8*, 1396–1405.

Hagemann, S., Günther, T., Dennemärker, J., Lohmüller, T., Brömme, D., Schüle, R., Peters, C., and Reinheckel, T. (2004). The human cysteine protease cathepsin V can compensate for murine cathepsin L in mouse epidermis and hair follicles. Eur. J. Cell Biol. *83*, 775–780.

Hartmann, H., Lübbers, B., Casaretto, M., Bautsch, W., Klos, A., and Köhl, J. (1993). Rapid quantification of C3a and C5a using a combination of chromatographic and immunoassay procedures. J Immunol Methods *166*, 35–44.

Van den Berg, C.W., Van Dijk, H., and Capel, P.J. (1989). Rapid isolation and characterization of native mouse complement components C3 and C5. J Immunol Methods *122*, 73–78.

Wang, G., Liszewski, M.K., Chan, A.C., and Atkinson, J.P. (2000). Membrane cofactor protein (MCP; CD46): isoform-specific tyrosine phosphorylation. J Immunol *164*, 1839–1846.

Wessels, M.R., Butko, P., Ma, M., Warren, H.B., Lage, A.L., and Carroll, M.C. (1995). Studies of group B streptococcal infection in mice deficient in complement component C3 or

C4 demonstrate an essential role for complement in both innate and acquired immunity. Proc Natl Acad Sci USA *92*, 11490–11494.

Weyand, N.J., Lee, S.W., Higashi, D.L., Cawley, D., Yoshihara, P., and So, M. (2006). Monoclonal antibody detection of CD46 clustering beneath Neisseria gonorrhoeae microcolonies. Infect Immun *74*, 2428–2435.
